# Supplementary material for: Analysis of CFTR Mutation Spectrum in Ethnic Russian Cystic Fibrosis Patients
Source: Genes (Basel). 2020 May 15;11(5):554. doi: 10.3390/genes11050554 (PMC7288340; doi:10.3390/genes11050554)
Supplement: Supplementary file 1 [file genes-11-00554-s001.zip › genes-774075-supplmentary-tableS3-14.05.2020 (1).docx]

Table S3. Clinical and demographic characteristics of CF patients with rare missense variants (according to RF CF Registry).

| **Genotype** | **Patient 1** | **Patient 2** | **Patient 3** | **Patient 4** | **Patient 5** |
| --- | --- | --- | --- | --- | --- |
| Allele 1 | c.1521_1523delCTT  (F508del) | c.54-5940_273+10250del21kb (CFTRdele2,3) | c.1521_1523delCTT  (F508del) | c.54-5940_273+10250del21kb (CFTRdele2,3) | c.1521_1523delCTT  (F508del) |
| Allele 2 | **c.650A>G (E217G)** | **c.358G>C (p.Ala120Pro)** | **c.1382G>A (p.Gly461Glu)** | **c.1382G>A (p.Gly461Glu))** | **c.1513A>C (p.Asn505His)** |
| **Basic characteristics** | | | | | |
| Gender | female | female | female | female | female |
| Age (yrs) | 4.47 | 4.63 | 15.95 | 4.59 | 1.32 |
| Age at diagnosis (yrs) | 0.14 | 0.11 | 8.81 | 0.02 | 0.05 |
| Height (cm) | 104 | 92 | 165 | 116 | 76 |
| Weight (kg) | 18.5 | 14.8 | 48.0 | 19.5 | 10.0 |
| **Clinical characteristics** | | | | | |
| CF Neonatal Screening | positive | positive | not done | positive | negative |
| Sweat chloride (mmol/l) | 63 | 95 | 117 | 117 | 122 |
| FVC (%) | - | - | 82.8 | - | - |
| FEV_1_ (%) | - | - | 58.0 | - | - |
| Meconium ileus | no | no | no | no | yes |
| Diabetes | no | no | no | no | no |
| Fecal elastase 1 (µg/g) | >200 | >200 | <200 | <200 | >200 |
| Liver disease | no | no | yes, without cirrhosis | no | no |
| Nasal polyposis | no | no | yes | no | no |
| **Bacterial flora characteristics** | | | | | |
| *S.aureus* | yes | no | no | yes | yes |
| *P.aeruginosa* | yes | no | no | yes | yes |
| *Achromobacte*r spp. | no | no | no | no | yes |
| *Stenotrophomonas* spp. | no | no | no | no | no |
| *B.cepacia* | no | no | no | no | no |

“no” – not identified

| **Genotype** | **Patient 6** | **Patient 7** | **Patient 8** | **Patient 9** | **Patient 10** |
| --- | --- | --- | --- | --- | --- |
| Allele 1 | c.1608delA (p.Asp537ThrfsX3) | c.1521_1523delCTT  (F508del) | c.1521_1523delCTT  (F508del) | c.1521_1523delCTT  (F508del) | c.1521_1523delCTT  (F508del) |
| Allele 2 | **c.1525G>C (p.Gly509Arg)** | **c.613C>A (p.Pro205Thr)** | **c.1352G>T (p.Gly451Val)** | **c.1589T>C (p.Ile530Thr)** | **c.3107C>A (p.Thr1036Asn)** |
| **Basic characteristics** | | | | | |
| Gender | female | male | female | male | female |
| Age (yrs) | 3.33 | 12.45 | 4.06 | 18.56 | 19.04 |
| Age at diagnosis (yrs) | 0.31 | 3.82 | 0.12 | 1.43 | 11.66 |
| Height (cm) | 87 | 154 | 102 | 164 | 162 |
| Weight (kg) | 10.5 | 48.7 | 15.7 | 62.0 | 48.0 |
| **Clinical characteristics** | | | | | |
| Diagnosis based on Neonatal Screening | positive | not done | positive | not done | not done |
| Sweat chloride (mmol/l) | 113 | 134 | 118 | 89 | 102 |
| FVC (%) | - | 90.2 | - | 87.3 | 97.6 |
| FEV_1_ (%) | - | 84.6 | - | 80.0 | 97.0 |
| Meconium ileus | yes | no | no | no |  |
| Diabetes | no | no | no | no | no |
| Fecal elastase 1 (µg/g) | <200 | not done | <200 | not done | not done |
| Liver disease | yes | no | no | no | no |
| Nasal polyposis | no | no | no | yes |  |
| **Bacterial flora characteristics** | | | | | |
| *S.aureus* | no | yes | yes | yes | yes |
| *P.aeruginosa* | yes | yes | no | no | no |
| *Achromobacte*r spp. | no | no | no | no | no |
| *Stenotrophomonas* spp. | no | no | no | no | no |
| *B.cepacia* | no | no | no | no | no |

“no” – not identified
