# Supplementary material for: Analysis of CFTR Mutation Spectrum in Ethnic Russian Cystic Fibrosis Patients
Source: Genes (Basel). 2020 May 15;11(5):554. doi: 10.3390/genes11050554 (PMC7288340; doi:10.3390/genes11050554)
Supplement: Supplementary file 1 [file genes-11-00554-s001.zip › genes-774075-supplementary-14.05.2020.docx]

**Supplementary Materials** to the manuscript “**Analysis of CFTR mutation spectrum in ethnic Russian CF patients**” by Nika V. Petrova, Natalya Y. Kashirskaya, Tatyana A. Vasilyeva, Elena I. Kondratyeva, Elena K. Zhekaite, Anna Y. Voronkova, Victoria D. Sherman, Varvara A. Galkina, Eugeny K. Ginter, Sergey I. Kutsev, Andrey V. Marakhonov and Rena A. Zinchenko

Table S1.. Frequencies of 33 variants of *CFTR* gene in the samples of 1384 ethnic Russians and in the CF patients from CFTR2 database [22].

| **No.** | **Variants** | **Ethnic Russian** | **CFTR**2 |  |
| --- | --- | --- | --- | --- |
|  |  | **%** | **%** | p |
| 1 | c.1521_1523delCTT (p.Phe508del, F508del) | 54.99 | 69.744 | <0.0001 |
| 2 | c.54-5940_273+10250del21kb (p.Ser18Arg*fsX16, CFTRdele2,3) | 7.59 | 0.294 | <0.0001 |
| 3 | c.2012delT (p.Leu671X, 2143delT) | 2.71 | 0.068 | <0.0001 |
| 4 | c.3718-2477C>T (3849+10kbC-T) | 2.35 | 0.815 | <0.0001 |
| 5 | c.2052_2053insA (p.Gln685ThrfsX4, 2184insA) | 2.25 | 0.232 | <0.0001 |
| 6 | c.3909C>G (p.Asn1303Lys, N1303K) | 1.74 | 1.581 | 0.5240 |
| 7 | c.1624G>T (p.Gly542X, G542X) | 1.60 | 2.542 | 0.0016 |
| 8 | c.274G>A (p.Glu92Lys, E92K) | 1.05 | 0.034 | <0.0001 |
| 9 | c.3846G>A (p.Trp1282X, W1282X) | 1.16 | 1.215 | 0.7735 |
| 10 | c.413_415dupTAC (p.Leu138dup; L138ins) | 1.12 | 0.014 | <0.0001 |
| 11 | c.3844T>C (p.Trp1282Arg, W1282R) | 0.76 |  |  |
| 12 | c.1397C>G (p.Ser466X, Ser466X) | 0.72 | 0.006 | <0.0001 |
| 13 | c.3691delT (p.Ser1231ProfsX4, 3821delT) | 0.69 | 0.004 | <0.0001 |
| 14 | c.1000C>T (p.Arg334Trp, R334W) | 0.69 | 0.302 | 0.0006 |
| 15 | c.262_263delTT (p.Leu88IlefsX22, 394delTT) | 0.54 | 0.216 | 0.0007 |
| 16 | c.3587C>G (p.Ser1196X, S1196X) | 0.51 | 0.013 | <0.0001 |
| 17 | c.3816_3817delGT (p.Ser1273LeufsX28, 3944delGT) | 0.43 |  |  |
| 18 | c.2657+5G>A (2789+5A>G) | 0.36 | 0.723 | 0.0339 |
| 19 | c.489+1G>T (621+1G>T) | 0.25 | 0.931 | 0.0003 |
| 20 | c.3140-16T>A (3272-16T>A) | 0.22 |  |  |
| 21 | c.1657C>T (p.Arg553X, R553X) | 0.18 | 0.931 | <0.0001 |
| 22 | c.1545_1546delTA (p.Tyr515X, 1677delTA) | 0.18 | 0.092 | 0.2338 |
| 23 | c.3535_3536insTCAA (p.Thr1179IlefsX17, 3667ins4) | 0.14 | 0.007 | <0.0001 |
| 24 | c.254G>A (p.Gly85Glu, G85E) | 0.14 | 0.434 | 0.0307 |
| 25 | c.472dupA (p.Ser158LysfsX5, 604insA) | 0.11 |  |  |
| 26 | c.2051_2052delAAinsG (p.Lys684SerfsX38, 2183AA>G) | 0.11 | 0.382 | 0.0301 |
| 27 | c.3475T>C (p.Ser1159Pro; S1159P) | 0.11 | 0.006 | <0.0001 |
| 28 | c.1040G>C (p.Arg347Pro, R347P) | 0.07 | 0.375 | 0.0146 |
| 29 | c.350G>A (p.Arg117His, R117H) | 0.04 | 1.305 | <0.0001 |
| 30 | c.1519_1521delATC (p.Ile507del, I507del) | - | 0.458 | <0.0001 |
| 31 | c.1585-1G>A (1717-1G>A) | - | 0.856 | <0.0001 |
| 32 | c.1652G>A (p.Gly551Asp, G551D) | - | 2.102 | <0.0001 |
| 33 | c.3476C>T (p.Ser1159Phe, S1159F) | - | 0.010 | 0.6150 |
|  |  | 82.78 | 77.07 |  |
|  | **Total** | 2768 | 142036 |  |

- - In all tested cases c.1397C>G (p.Ser466X, Ser466X) variant occurred in *cis* with c.3209G>A (p.Arg1070Gln, R1070Q) variant yielding the complex allele c.[1397C>G; 3209G>A] (p.[Ser466X; Arg1070Gln], Ser466X-R1070Q).

Table S2. Genotypes and *CFTR* gene variants in 154 Russian patients tested for 33 common *CFTR* variants.

| **Genotype** | **Number of patients** | **Variant** | **Number of alleles** | **%** |
| --- | --- | --- | --- | --- |
| c.[1521_1523delCTT];[?] (F508del/n) | 98 | c.1521_1523delCTT (F508del) | 98 | 31.8 |
| c.[54-5940_273+10250del21kb];[?] (CFTRdele2,3/n) | 20 | c.54-5940_273+10250del21kb (CFTRdele2,3) | 20 | 6.5 |
| c.[2012delT];[?] (2143delT/n) | 4 | c.2012delT (2143delT) | 4 | 1.3 |
| c.[1624G>T];[?] (G542X/n) | 3 | c.1624G>T (G542X) | 3 | 1.0 |
| c.[2052_2053insA];[?] (2184insA/n | 2 | c.2052_2053insA (2184insA) | 2 | 0.6 |
| c.[3909C>G];[?] (N1303K/n) | 2 | c.3909C>G (N1303K) | 2 | 0.6 |
| c.[1397C>G];[?] (S466X/n) | 3 | c.1397C>G (S466X) | 3 | 0.9 |
| c.[411_412insCTA];[?] (L138ins/n) | 2 | c.411_412insCTA (L138ins) | 2 | 0.6 |
| c.[3844T>C];[?] (W1282R/n) | 2 | c.3844T>C (W1282R) | 2 | 0.6 |
| c.[3475T>C];[?] (S1159P/n) | 1 | c.3475T>C (S1159P) | 1 |  |
| c.[1657C>T];[?] (R553X/n) | 1 | c.1657C>T (R553X) | 1 |  |
| c.[489+1G>T];[?] (621+1G>T/n) | 2 | c.489+1G>T (621+1G>T) | 2 | 0.6 |
| c.[3718-2477C>T];[?] (3849+10kbC-T/n) | 2 | c.3718-2477C>T (3849+10kbC->T) | 2 | 0.6 |
| c.[3535_3536insTCAA];[?] (3667ins4/n) | 1 | c.3535_3536insTCAA (3667ins4) | 1 | 0.3 |
| c.[3691delT];[?] (3821delT/n) | 1 | c.3691delT (3821delT) | 1 | 0.3 |
| c.[?];[?] (n/n) | 10 | c.? (unidentified) | 164 | 53.2 |
| Total | 154 |  | 308 |  |

Table S3. Clinical and demographic characteristics of CF patients with rare missense variants.

| **Genotype** | **Patient 1** | **Patient 2** | **Patient 3** | **Patient 4** | **Patient 5** |
| --- | --- | --- | --- | --- | --- |
| Allele 1 | c.1521_1523delCTT  (F508del) | c.54-5940_273+10250del21kb (CFTRdele2,3) | c.1521_1523delCTT  (F508del) | c.54-5940_273+10250del21kb (CFTRdele2,3) | c.1521_1523delCTT  (F508del) |
| Allele 2 | **c.650A>G (E217G)** | **c.358G>C (p.Ala120Pro)** | **c.1382G>A (p.Gly461Glu)** | **c.1382G>A (p.Gly461Glu))** | **c.1513A>C (p.Asn505His)** |
| **Basic characteristics** | | | | | |
| Gender | female | female | female | female | female |
| Age (yrs) | 4.47 | 4.63 | 15.95 | 4.59 | 1.32 |
| Age at diagnosis (yrs) | 0.14 | 0.11 | 8.81 | 0.02 | 0.05 |
| Height (cm) | 104 | 92 | 165 | 116 | 76 |
| Weight (kg) | 18.5 | 14.8 | 48.0 | 19.5 | 10.0 |
| BMI (kg/m^2^) |  |  |  |  |  |
| Clinical characteristics |  |  |  |  |  |
| CF Neonatal Screening | positive | positive | not done | positive | negative |
| Sweat chloride (mmol/l) | 63 | 95 | 117 | 117 | 122 |
| FVC (%) | - | - | 82.8 | - | - |
| FEV_1_ (%) | - | - | 58.0 | - | - |
| Meconium ileus | no | no | no | no | yes |
| Diabetes | no | no | no | no | no |
| Fecal elastase 1 (µg/g) | >200 | >200 | <200 | <200 | >200 |
| Liver damage | no | no | yes, without cirrhosis | no | no |
| Nasal polyposis | no | no | yes | no | no |
| **Bacterial flora characteristics** | | | | | |
| *S.aureus* | yes | no | no | yes | yes |
| *P.aeruginosa* | yes | no | no | yes | yes |
| *Achromobacte*r spp. | no | no | no | no | yes |
| *Stenotrophomonas* spp. | no | no | no | no | no |
| *B.cepacia* | no | no | no | no | no |

| **Genotype** | **Patient 6** | **Patient 7** | **Patient 8** | **Patient 9** | **Patient 10** |
| --- | --- | --- | --- | --- | --- |
| Allele 1 | c.1608delA (p.Asp537ThrfsX3) | c.1521_1523delCTT  (F508del) | c.1521_1523delCTT  (F508del) | c.1521_1523delCTT  (F508del) | c.1521_1523delCTT  (F508del) |
| Allele 2 | **c.1525G>C (p.Gly509Arg)** | **c.613C>A (p.Pro205Thr)** | **c.1352G>T (p.Gly451Val)** | **c.1589T>C (p.Ile530Thr)** | **c.3107C>A (p.Thr1036Asn)** |
| **Basic characteristics** | | | | | |
| Gender | female | male | female | male | female |
| Age (yrs) | 3.33 | 12.45 | 4.06 | 18.56 | 19.04 |
| Age at diagnosis (yrs) | 0.31 | 3.82 | 0.12 | 1.43 | 11.66 |
| Height (cm) | 87 | 154 | 102 | 164 | 162 |
| Weight (kg) | 10.5 | 48.7 | 15.7 | 62.0 | 48.0 |
| BMI (kg/m^2^) |  |  |  |  |  |
| Clinical characteristics |  |  |  |  |  |
| Diagnosis based on Neonatal Screening | positive | not done | positive | not done | not done |
| Sweat chloride (mmol/l) | 113 | 134 | 118 | 89 | 102 |
| FVC (%) | - | 90.2 | - | 87.3 | 97.6 |
| FEV_1_ (%) | - | 84.6 | - | 80.0 | 97.0 |
| Meconium ileus | yes | no | no | no |  |
| Diabetes | no | no | no | no | no |
| Fecal elastase 1 (µg/g) | <200 | not done | <200 | not done | not done |
| Liver disease | yes (1) | no | no | no | no |
| Nasal polyposis | no | no | no | yes |  |
| **Bacterial flora characteristics** | | | | | |
| *S.aureus* | no | yes | yes | yes | yes |
| *P.aeruginosa* | yes | yes | no | no | no |
| *Achromobacte*r spp. | no | no | no | no | no |
| *Stenotrophomonas* spp. | no | no | no | no | no |
| *B.cepacia* | no | no | no | no | no |
